# Accumulation of citrullinated glial fibrillary acidic protein in a mouse model of bile duct ligation-induced hepatic fibrosis

**DOI:** 10.1371/journal.pone.0201744

**Published:** 2018-08-02

**Authors:** Sung-Eun Kim, Ji Won Park, Mo-Jong Kim, Byungki Jang, Yong-Chul Jeon, Hee-Jun Kim, Akihito Ishigami, Hyoung Su Kim, Ki Tae Suk, Dong Joon Kim, Choong Kee Park, Eun-Kyoung Choi, Myoung-Kuk Jang

**Affiliations:** 1 Department of Internal Medicine, Hallym University Sacred Heart Hospital, College of Medicine, Hallym University, Anyang, Republic of Korea; 2 Department of Biomedical Gerontology, Graduate School of Hallym University, Anyang, Republic of Korea; 3 Ilsong Institute of Life Science, Hallym University, Anyang, Republic of Korea; 4 Molecular Regulation of Aging, Tokyo Metropolitan Institute of Gerontology, Tokyo, Japan; 5 Department of Internal Medicine, Kangdong Sacred Heart Hospital, College of Medicine, Hallym University, Seoul, Republic of Korea; 6 Department of Internal Medicine, Chuncheon Sacred Heart Hospital, College of Medicine, Hallym University, Chuncheon, Republic of Korea; Vrije Universiteit Brussel, BELGIUM

## Abstract

Hepatic stellate cells (HSCs) play pivotal roles in hepatic fibrosis as they synthesize glial fibrillary acidic protein (GFAP), which is increased in activated HSCs. GFAP-expressing HSCs and myofibroblasts accumulate in and around hepatic fibrosis lesions. Peptidylarginine deiminase 2 (PAD2) is responsible for the citrullination of GFAP (cit-GFAP). However, the involvement of PAD2 and cit-GFAP in hepatic fibrosis remains unclear. To determine the expression of PAD2 and cit-GFAP in hepatic fibrosis, C57BL/6 mice underwent bile duct ligation (BDL) or a sham operation. In BDL livers, the expression of PAD2 and its enzyme activity were significantly increased compared with controls. In addition, PAD2-postitive cells were rarely observed in only the portal vein and the small bile duct in sham-operated livers, whereas an increased number of PAD2-positive cells were detected in the bile duct and Glisson’s sheath in BDL livers. Interestingly, PAD2 was colocalized with α-SMA-positive cells and CK19-positive cells in BDL livers, indicating upregulated PAD2 in activated HSCs and portal fibroblasts of the livers of BDL mice. We also found that citrullinated proteins were highly accumulated in the livers of BDL mice compared with controls. Moreover, the expression level of GFAP and the amount of cit-GFAP were higher in BDL livers than in control livers. In correlation with PAD2 localization, cit-GFAP was observed in α-SMA-positive and CK19-positive cells in the livers of BDL mice. These results suggest that the increased expression and activation of PAD2 along with increased citrullinated proteins, specifically cit-GFAP, may play important roles in the pathogenesis of hepatic fibrosis.

## Introduction

Hepatic fibrosis is induced by various etiologies of chronic hepatitis C virus (HCV) infection, chronic B virus infection, alcoholism, nonalcoholic fatty liver disease, autoimmune diseases and cholestasis, among others [1–3]. Hepatic fibrosis is defined as the result of excessive deposition of extracellular matrix, which disrupts the normal architecture of the liver [2]. Although hepatic fibrosis is considered to be a reversible process at the initial stage [1, 4], hepatic fibrosis progresses to advanced stages, such as cirrhosis or hepatocellular carcinoma, which cause impaired liver function and subsequent morbidity and mortality worldwide [1–3].

Activation of hepatic stellate cells (HSCs) are believed to play a major role in hepatic fibrosis and results in conversion to myofibroblasts [5, 6], which produce extracellular matrix proteins such as collagens. Transformed HSC-derived myofibroblasts from quiescent HSCs by fibrotic stimuli express increased cytoskeletal proteins such as α-smooth muscle actin (α-SMA), vimentin, and desmin [5]. Alpha-SMA is a specific marker for well-differentiated myofibroblasts [7]. Quiescent HSCs store retinoids and synthesize glial fibrillary acidic protein (GFAP) in normal liver [8].

GFAP, a type III intermediate filament (IF) protein responsible for the cytoskeletal structure of glial cells, is critical in maintaining the IF network of activated astrocytes, with processes that develop thickened and elongated shapes [9]. GFAP is also noted in HSC-derived myofibroblasts [10] and in activated rodent HSCs with a gradual loss after liver injury suggesting GFAP as a marker for quiescent cells in rodents [11]. In contrast, recent studies have reported that GFAP-expressing HSCs and myofibroblasts accumulate in and around hepatic fibrosis lesions and that the amount of GFAP increases with the progression of hepatic fibrosis [12, 13]. These findings suggest that the level GFAP expression in HSCs is related to the fibrosis progression and the disease severity.

Citrullination is a posttranslational modification that abolishes the positive charges of arginine residues by conversion to citrulline residues via calcium-dependent peptidylarginine deiminases (PADs), leading to significant alterations in protein structure and function [14–17]. PAD represents 5 different isoforms, type 1–4 and type 6, which have distinct substrate and tissue specificity [18]. Among them, PAD2 shows ubiquitous distribution in mammalian tissues, such as in muscle, dermis, spleen, and hematopoietic cells [18, 19]. Although PAD2 normally exists as an inactive form, it becomes active and citrullinates cellular proteins when the intracellular calcium balance is upset during neurodegenerative changes [20].

GFAP was identified as a highly susceptible substrate of PAD2 [21], and abnormal accumulation of citrullinated GFAP (cit-GFAP) was found in neurodegenerative diseases, including Alzheimer’s disease and prion disease [15, 21, 22]. However, the expression and role of cit-CFAP in hepatic fibrosis have not been reported. For the first time, we show the accumulation of cit-GFAP and upregulated PAD2 in bile duct ligation (BDL)-induced hepatic fibrosis.

## Materials and methods

### Animal models

C57BL/6 mice (9–10 weeks old male, Raon Bio, Inc., Korea) were purchased and housed in specific pathogen-free conditions in the animal facility of the Ilsong Institute of Life Science, Anyang, Korea. Mice were randomly assigned to two groups: a sham-operated control group and a BDL group. Before the operation, mice were injected intraperitoneally with 200 mg/kg tribromoethanol, followed by a midline abdominal incision and isolation and double-ligation of the common bile duct. In sham-operated controls, the common bile duct was manipulated but not ligated. Three weeks after surgery, the mice were euthanized using CO_2,_ and their livers were extracted under anesthesia. For the treatment of thioacetamide (TAA), mice were injected intraperitoneally three times per week with TAA (Wako Pure Chemical Industries, Osaka, Japan) dissolved in 0.9% NaCl at a dose of 100 mg/kg body weight. Six mice were examined each at weeks 4 after the first injection. Six control mice were received an equal volume of 0.9% NaCl in the same manner, and control livers were extracted each at weeks 4 under anesthesia.

Animal experiments and research protocols were approved by the Hallym Medical Center Institutional Animal Care and Use Committee (HMC2017-1-0622-20).

### Western blot analysis

Briefly, equal amounts of protein (50 μg/lane) were subjected to 10 or 12% SDS-PAGE and transferred to polyvinylidene difluoride (PVDF) membranes (Millipore, Billerica, MA) using an electrotransfer system (Bio-Rad, Hercules, CA). The membranes were blocked with 5% nonfat dry milk in PBST (8 mM Na_2_HPO_4_, 2 mM KH_2_PO_4_, 138 mM NaCl, 2.7 mM KCl, pH 7.4, 0.1% Tween 20) for 1 hour at room temperature (RT). The membranes were then incubated with mouse monoclonal anti-PAD2 [23], mouse monoclonal anti-F95 (anti-peptidyl-citrulline antibody, Millipore), rabbit polyclonal anti-GFAP (Abcam, Cambridge UK), mouse monoclonal citrullinated GFAP (CTGF-122R and CTGF-1221)[22] and rabbit polyclonal glyceraldehyde-3-phosphate dehydrogenase (GAPDH) (Santa Cruz Biotechnology, Santa Cruz, CA) antibodies in PBST overnight at 4°C. The membranes were incubated with the appropriate secondary antibodies (Enzo Life Sciences, Farmingdale, NY) conjugated to horseradish peroxidase for 2 hours at room temperature (RT). Chemiluminescent signals were visualized by using a chemiluminescent substrate (ATTO, Tokyo, Japan) and a LAS-4000 Bio imaging Analyzer System (GE Healthcare Life Science, Piscataway, NJ, USA).

### Quantitative real-time polymerase chain reaction (qPCR)

Total RNA was extracted from liver tissues of sham-operated and BDL mice using an RNA purification Kit (GeneAll, Seoul, Republic of Korea) according to the manufacturer’s protocols. Complementary DNA was generated using AMV reverse transcriptase (Promega, Madison, WI, USA) according to the manufacturer’s instructions. qPCR was performed using primers specific for SMA, collagen type I (Col1a1) and GAPDH. Data were normalized to GAPDH. The primers used for qPCR are listed in Table 1.

**Table 1:** Oligo nucleotide sequences used for quantitiative RT-PCR.

Gene	Sequence (forward/reverse)
Col1a1	5’-GCTCCTCTTAGGGGCCACT-3’
	5’-ATTGGGGACCCTTAGGCCAT-3’
α-SMA	5’-AAGCCCAGCCAGTCGCTGTCA-3’
	5’-GAAGCCGGCCTTACAGAGCCC-3’
GAPDH	5’-TGGCCTTCCGTGTTCCTA-3’
	5’-GAGTTGCTGTTGAAGTCGCA-3’

Col1a1, collagen type I; α-SMA, alpha-smooth muscle actin; GAPDH, glyceraldehyde-3phosphate dehydrogenase

### Measurement of PAD2 activity

PAD2 activity was measured as previously described [24]. Briefly, 400 μg of liver tissues from sham-operated and BDL mice were incubated with the reaction mixture (100 mM Tris-HCl, pH 7.5, 10 mM CaCl_2_, 5 mM dithiothreitol with or without 10 mM benzoyl-L-arginine ethyl ester (Sigma) at 37°C for 3 hours. The reaction was stopped by adding final 1 mol/L perchloric acid. Samples were cooled on ice for 20 minutes and then centrifuged at 13,500 rpm for 5 minutes at RT. Aliquots of 120 μl of supernatant were mixed with 380 μl of H_2_O and 500 μl of color developing reagents and incubated at 95°C for 10 minutes. The samples were cooled at RT, and then, the absorbance was monitored at 534 nm by an enzyme-linked immunosorbent assay reader (VersaMax; Molecular Devices, Sunnyvale, CA).

### Histology and immunohistochemistry

Six-micrometer-thick paraffin-embedded liver sections of sham-operated and BDL mice were stained with hematoxylin and eosin and Sirius red for histological analysis. Immunohistochemistry of PAD2, GFAP and citrullinated proteins was evaluated with rabbit polyclonal anti-PAD2 (Proteintech, Chicago, IL), goat polyclonal anti-GFAP (Santa Cruz Biotechnology, Santa Cruz, CA, USA) and mouse monoclonal anti-F95 (anti-peptidyl-citrulline; Millipore) antibodies.

### Double immunohistochemical staining

For characterization of the cellular localization of PAD2 and cit-GFAP, double immunohistochemical staining was performed using well-established markers for different hepatic cells: α-SMA (a marker for HSCs), cytokeratin (CK) 19 (a marker for cholangiocytes and portal fibroblasts), hepatic sinusoidal endothelial cell antibody (SE-1), F4/80 (a marker for hepatic macrophage). The liver sections (6 μm thick) were deparaffinized with xylene and hydrated in a graded ethanol series. The sections were blocked with 10% goat serum in PBST for 1 hour at RT and then incubated with rabbit polyclonal anti-PAD2 (Proteintech) with mouse monoclonal antibody for α-SMA (Dako, Copenhagen, Denmark) or rat polyclonal anti-CK 19 (Troma III; Developmental Studies Hybridoma Bank, University of Iowa) in PBST overnight at 4°C. Other liver sections were incubated with CTGF-122R monoclonal antibody with goat polyclonal anti-GFAP (Santa Cruz Biotechnology), mouse monoclonal anti-SMA, rat polyclonal anti-CK19, rat polyclonal antibody for SE-1 (Novus biological), or rat monoclonal anti-F4/80 (Abcam) overnight at 4°C. After rinsing with PBST, the sections were treated with Alexa Fluor 488 goat anti-mouse or rat and Alexa Fluor 568 goat anti-rabbit (Molecular Probes, Carlsbad, CA) in PBST for 1 hour at RT and then mounted using antifade mounting medium. Images were obtained using a confocal laser scanning microscope (LSM 700; Carl Zeiss, Oberkochen, Germany).

### Statistical analyses

All obtained data were expressed as the means ± standard deviation (SD). Differences between two groups were compared using two-tailed unpaired Student’s *t*-test. *P* values less than 0.05 were considered statistically significant. Statistical significance was defined as *P <0.05, **P <0.01, and ***P <0.001.

## Results

### Generation of BDL-induced hepatic fibrosis mouse model

**Fig 1:**
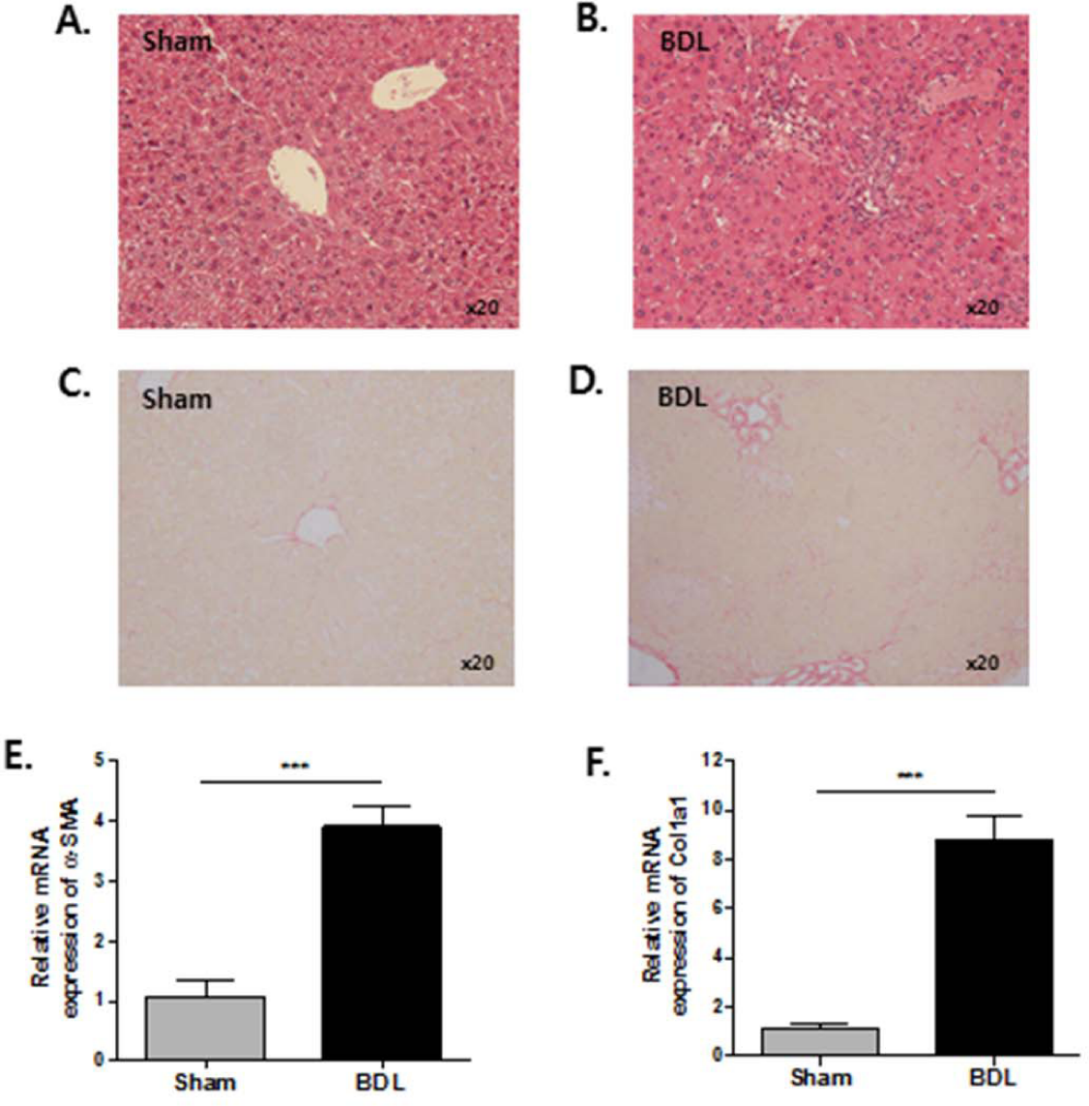
Development of hepatic fibrosis after BDL at week 3. (A) Liver tissues from sham-operated controls did not show any hepatic inflammation or bile duct injury by hematoxylin and eosin staining. (B) Hepatic inflammation and expansion of portal tract tracts with bile duct proliferation and periportal fibrosis was noted in the livers of BDL mice. (C) Collagen deposition was not observed in liver tissues from sham-operated controls by Sirius red staining. (D) Collagen deposition was observed around the bile duct and fibrous septa in the livers of BDL mice. (E) mRNA levels of α-SMA and Col1a1 were analyzed by qPCR. (n = 9). ***P <0.001.

To determine the expression level and enzyme activity of PAD2 in hepatic fibrosis, we developed an experimental mouse model of hepatic fibrosis by conducting BDL operation using C57BL/6 mice. By 3 weeks after the operation, hepatic inflammation had developed around the bile duct in the liver parenchyma of BDL mice controls (Fig 1B) compared with sham-operated controls (Fig 1A). Bridging fibrosis was also observed from the central veins to the portal area with increased deposition of collagen in the livers of BDL mice (Fig 1D) compared with sham-operated controls (Fig 1C). In addition, we measured mRNA expression of α-SMA and Col1a1 to define the degree of hepatic fibrosis. The expression levels of α-SMA and Col1a1 mRNAs were significantly increased in the livers of BDL mice but not in the livers of sham-operated controls (Fig 1E and 1F). These results showed that hepatic fibrosis was well developed in our BDL mouse model.

### Upregulation of PAD2 expression and elevated PAD2 activity in the livers of BDL mice

**Fig 2:**
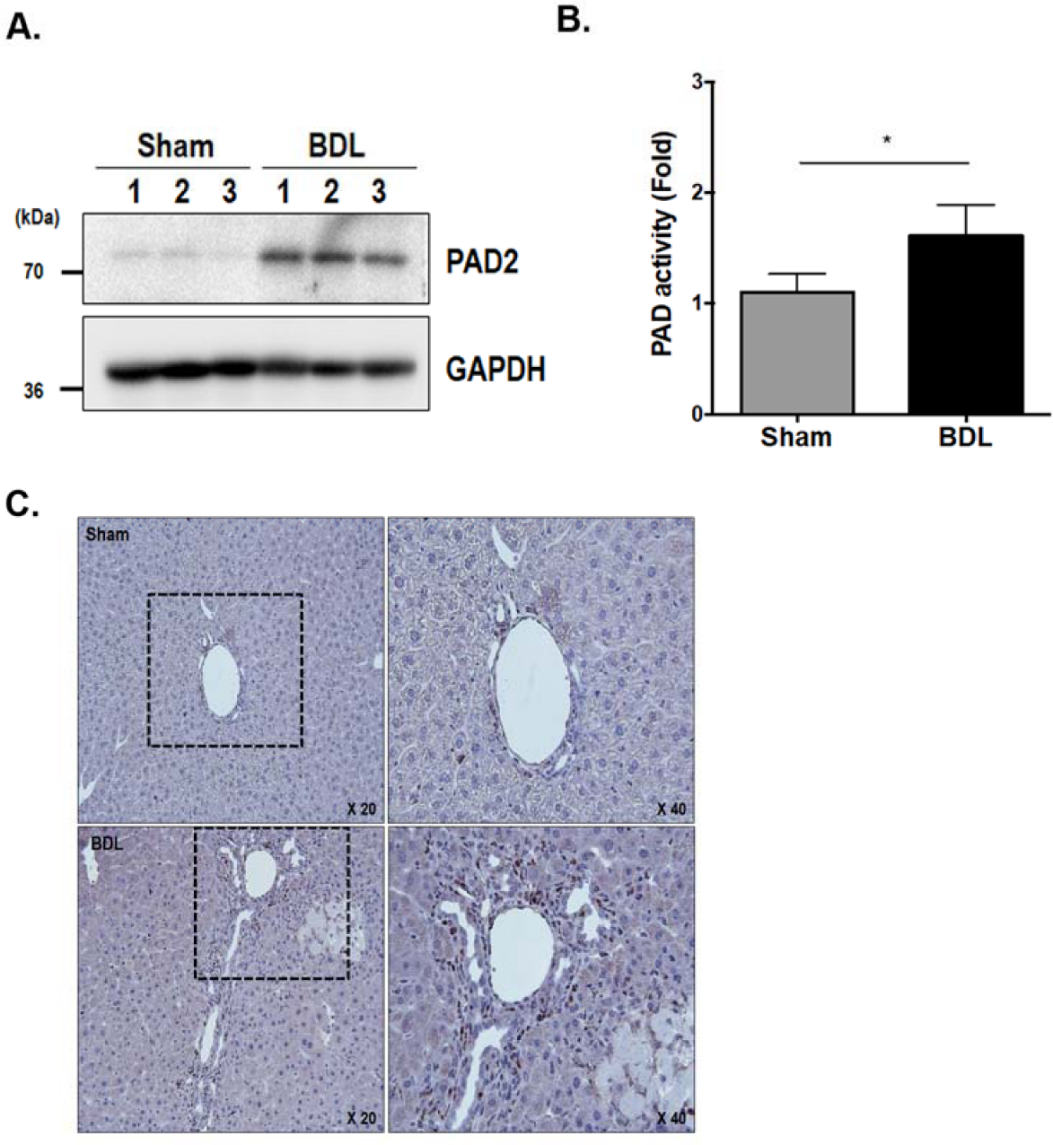
Comparison of PAD2 expression levels in livers from sham-operated control and BDL mice. (A) The expression level of PAD2 protein in the liver tissues of control and BDL mice was analyzed by western blot using monoclonal anti-PAD2 antibody. (B) Densitometric analysis of PAD2 activity demonstrated that the liver tissue of BDL mice (n = 9) showed increased PAD2 activity compared with controls (n = 9). *P <0.05. (C) Immunohistochemical staining of PAD2 in liver tissues from controls and BDL mice. In BDL liver, an increased number of PAD2-postive cells was located in the bile duct and Glisson’s sheath.

Next, to determine the involvement of PAD activation in hepatic fibrosis, we investigated the expression level and enzyme activity of PAD2 in the livers of BDL mice. The expression level of PAD2 was increased in the livers of BDL mice compared with sham-operated controls (Fig 2A). The enzyme activity of total PAD was also significantly higher in BDL livers than in sham-operated controls (Fig 2B). In sham-operated livers, PAD2-postive cells were only observed in some cells around the portal vein and the small bile duct (Fig 2C). In contrast, accumulated PAD2-postive cells were located in the bile duct and Glisson’s sheath in the BDL livers. Moreover, PAD2-positive cells were observed in the developing fibrotic lesion (Fig 2C).

We also observed similar results using the livers of TAA-treated mice compared to controls (S1 Fig). These results suggest that increased PAD2 expression together with its enzyme activity is involved in the development of hepatic fibrosis.

### Characterization of PAD2 localization in the livers of BDL-induced mice

**Fig 3:**
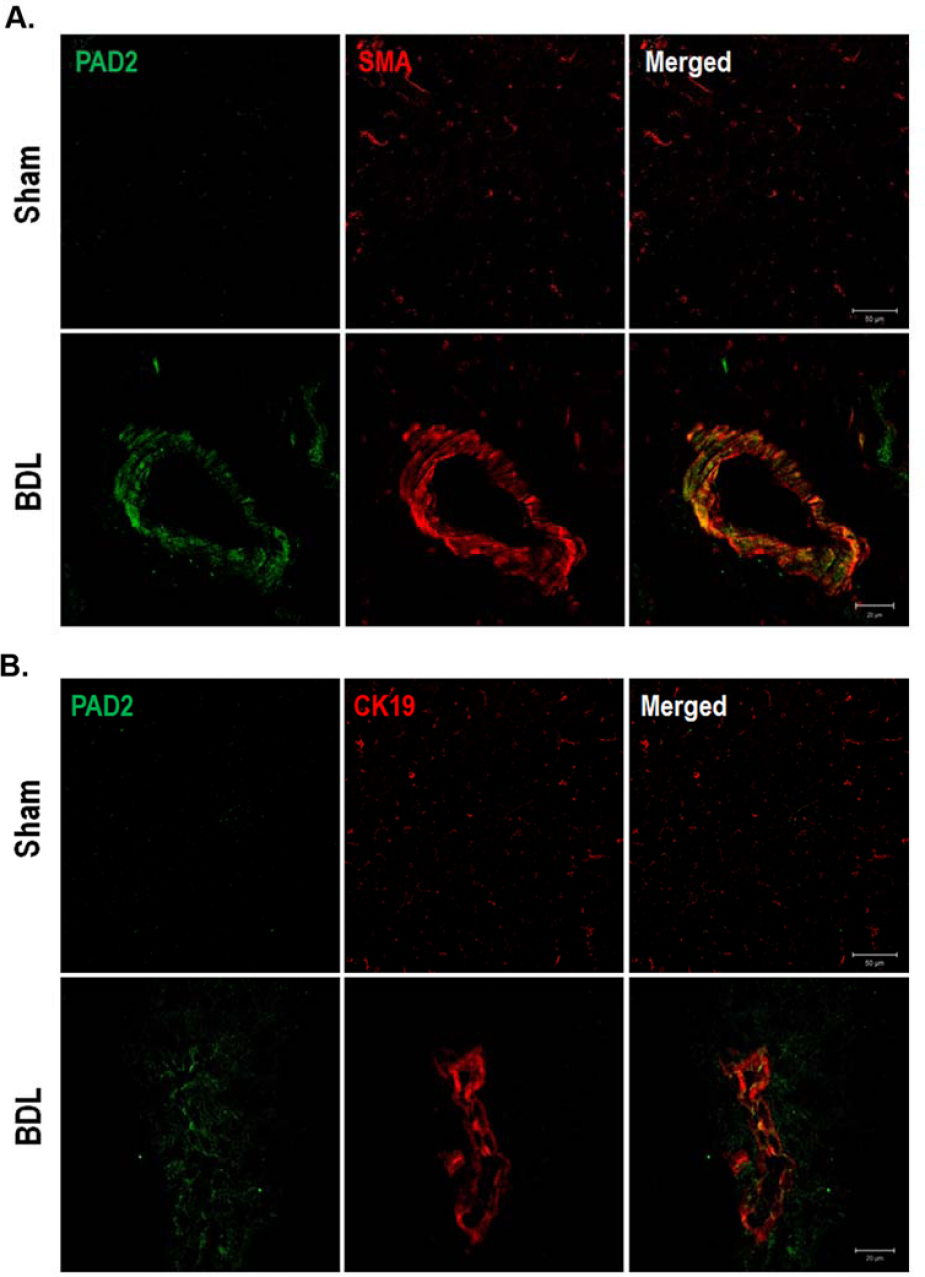
Double immunofluorescence of PAD2 and α-SMA or CK19 in livers from sham-operated control and BDL mice. Cryosections were examined under a confocal microscope. (A) PAD2 immunoreactivity was more intense in the livers of BDL mice than in the livers of sham-operated controls. Alpha-SMA immunoreactivity was also more intense in the livers of BDL mice than in the livers of controls. PAD2 was mainly colocalized with α-SMA-positive cells in and around the bile duct area (B) CK19 immunoreactivity was more intense in the livers of BDL mice than in the livers of controls. PAD2 was also colocalized with CK19-positive cells in and around the bile duct area.

To confirm the increased PAD2 expression and to determine the cellular localization of PAD2, double immunofluorescence analysis was performed in the livers from sham-operated and BDL mice. PAD2 immunoreactivity was more intense in the livers of the hepatic fibrosis model than in the livers of the sham-operated controls (Fig 3A and 3B). To further characterize the localization of PAD2, we performed double immunofluorescence analysis using α-SMA and CK19, which are representative markers for activated HSCs/portal fibroblasts and biliary epithelial/hepatic progenitor cells, respectively. Interestingly, PAD2 was mainly colocalized with α-SMA-positive cells and CK19-positive cells in the liver of BDL mice. We found similar results using the livers from TAA-induced mice although the intensity of colocalization of PAD2 with α-SMA or CK19 was less than the livers of BDL-induced hepatic fibrosis mice (S2 Fig). These results suggest that PAD2 expression is upregulated and intensely localized in activated HSCs and portal fibroblasts of the livers of BDL mice.

### Increased citrullinated proteins in BDL-induced mouse hepatic fibrosis

**Fig 4:**
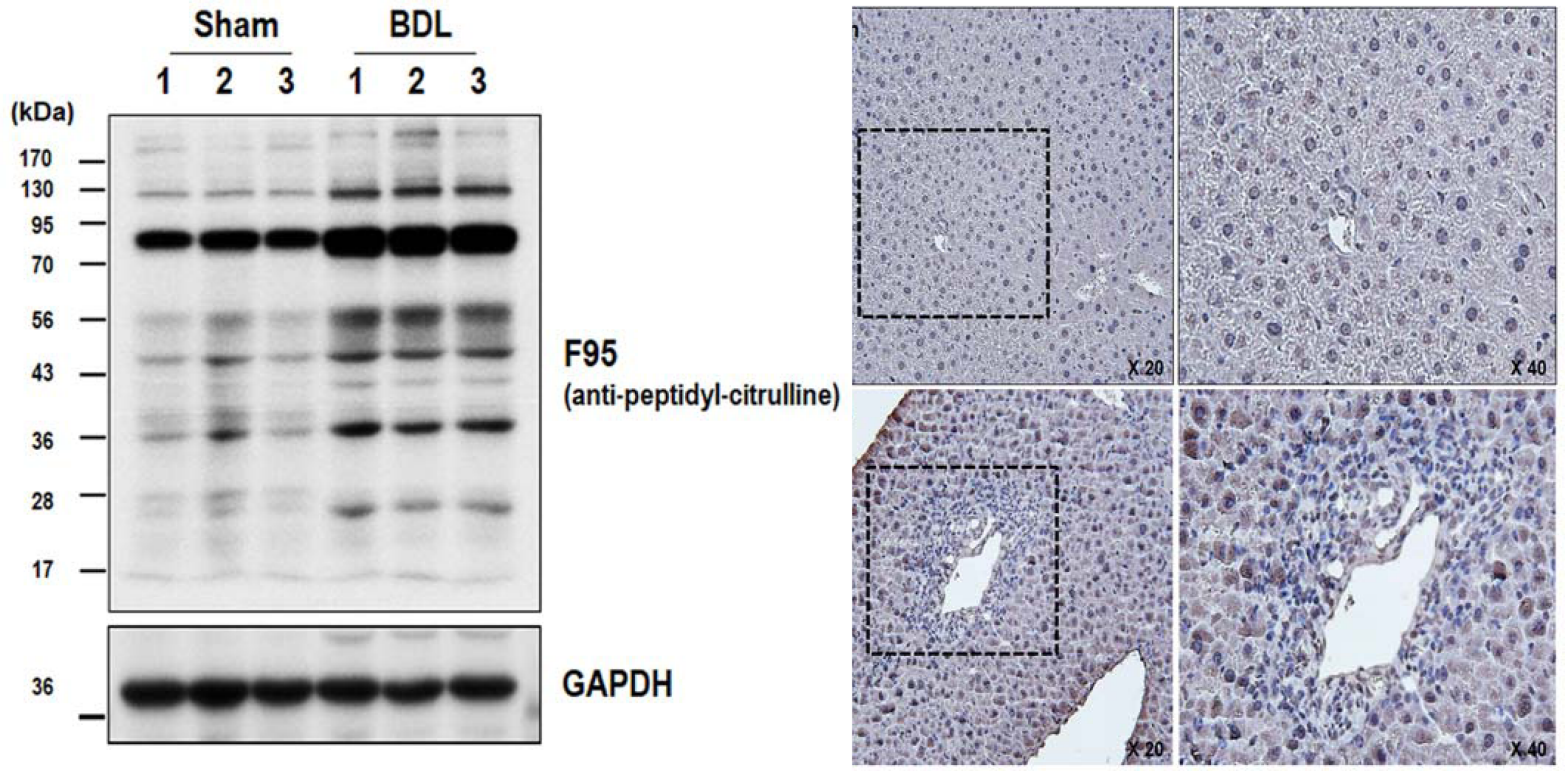
Detection of citrullinated proteins in the livers of sham-operated control and BDL mice. (A) The expression level of citrullinated protein in the liver tissues of control and BDL mice was analyzed by western blot using F95 antibody. Note that citrullinated proteins were increased in the liver tissues of BDL mice compared with controls. (B) Immunohistochemical staining of citrullinated proteins in liver tissues from controls and BDL mice. Citrullinated proteins were more prominent in the livers of BDL mice.

Because citrullination is regulated by PAD expression and its enzyme activity, which were increased in BDL livers, we next performed western blot analysis to examine the citrullinated proteins in the livers of sham-operated and BDL mice. Citrullinated proteins of BDL livers were markedly increased compared with sham control livers (Fig 4A). Most bands from 10 kDa to 130 kDa were increased in the livers of BDL mice compared with controls. Next, we conducted an immunohistochemical analysis of citrullinated proteins in the livers of sham-operated and BDL mice. As shown in Fig 4B, citrullinated proteins were more prominent in the livers of BDL mice compared with controls. In addition, similar results were found in the livers from TAA-induced mice although the amount of citrullinated proteins was less than the one in BDL livers (S1 Fig). These results indicate that upregulation of PAD2 expression and its activity are responsible for the increased citrullinated proteins in the livers with hepatic fibrosis.

### Increased GFAP and cit-GFAP expression in BDL-induced mouse hepatic fibrosis

**Fig 5:**
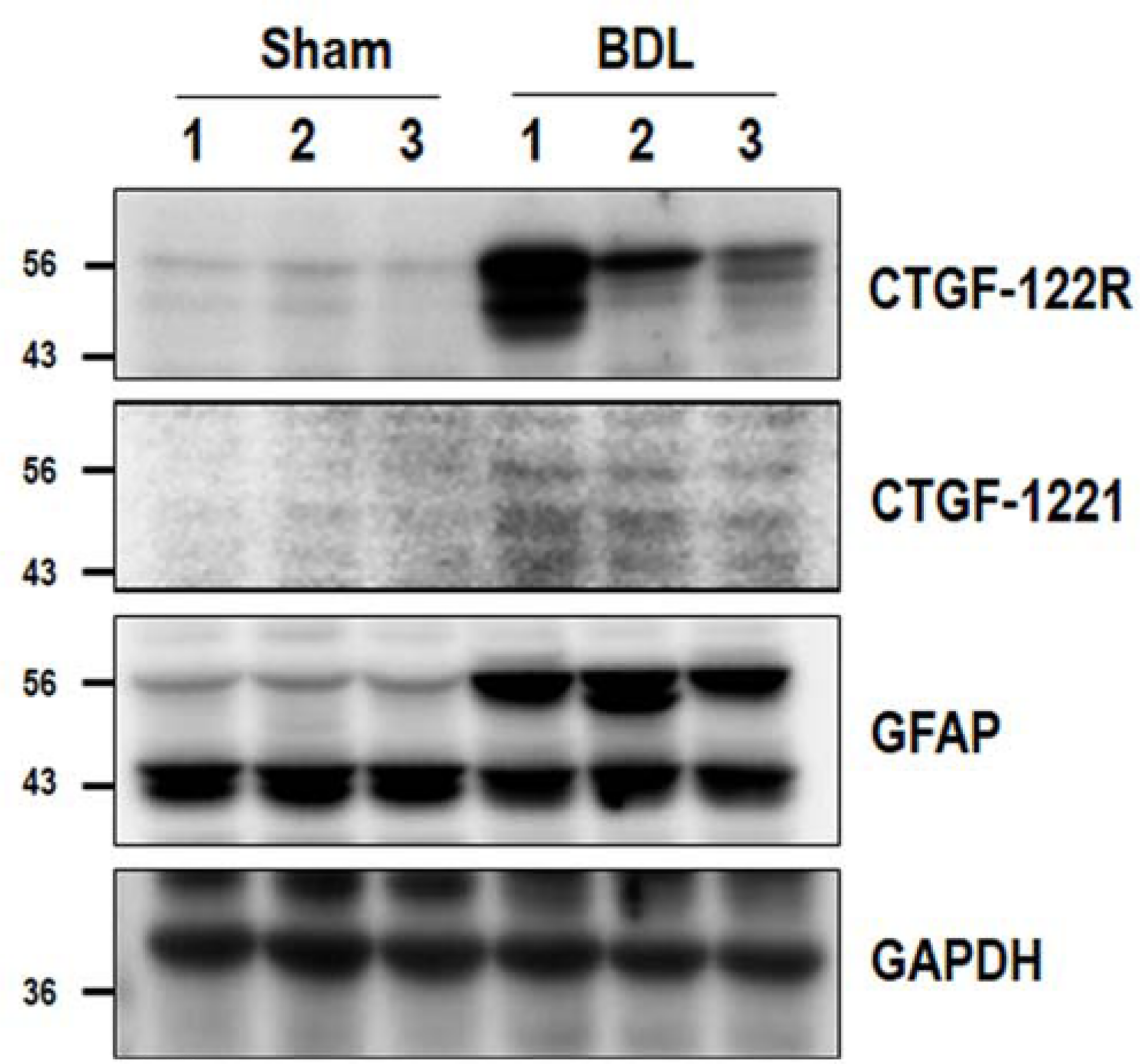
Detection of GFAP and cit-GFAP in livers from sham-operated control and BDL mice. The expression levels of GFAP and cit-GFAP in the liver tissues of control and BDL mice were analyzed by western blot using rabbit polyclonal anti-GFAP (Abcam, Cambridge UK) and citrullinated GFAP (CTGF-122R and CTGF-1221) antibodies. Note that GFAP and cit-GFAP were increased in the liver tissues of BDL mice compared with controls.

Because we observed significant increases in citrullinated proteins in BDL livers, we further investigated changes in the expression of GFAP, which is a highly susceptible substrate of PAD2, and the abnormal accumulation of cit-GFAP using western blot analysis. As shown in Fig 5, GFAP and cit-GFAP (CTGF-122R and CTGF-1221) in BDL livers were markedly increased compared with sham-operated livers. Among the bands of citrullinated proteins, the 56 kDa band of citrullinated proteins was correlated with the GFAP band. Accumulated cit-GFAP was clearly detectable in the livers of BDL mice compared with controls. GFAP and cit-GFAP (CTGF-122R) in TAA-treated livers were slightly increased compared with controls (S1 Fig). These results suggest that the higher expression of GFAP and the accumulation of cit-GFAP are responsible for the pathogenesis of hepatic fibrosis.

### Characterization of cit-GFAP localization in the livers of BDL mice

**Fig 6:**
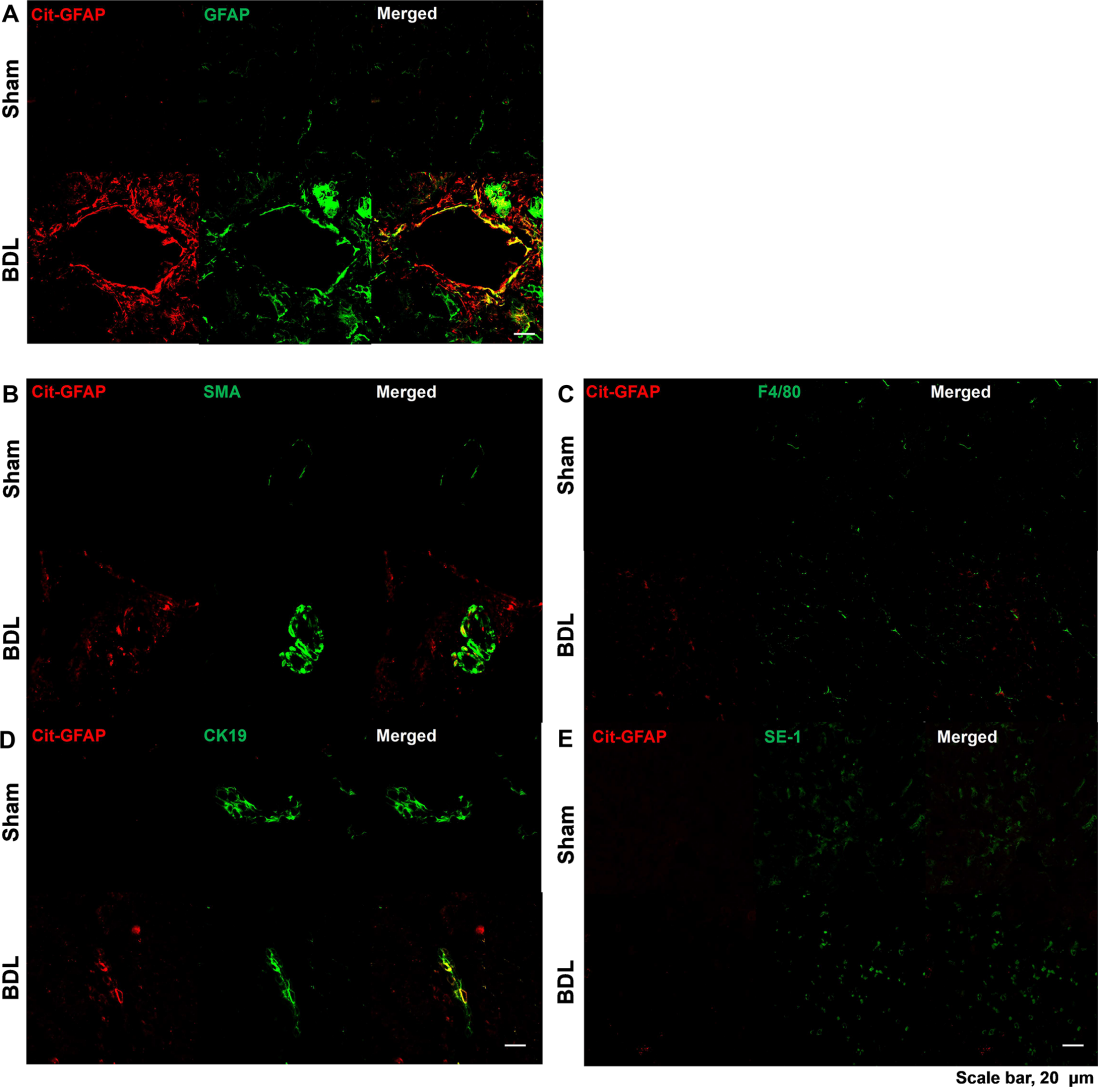
Double immunofluorescence of cit-GFAP (CTGF-122R) with GFAP (A), α-SMA (B), F4/80 (C), CK19 (D) or SE-1 (E) in the livers of sham-operated control and BDL mice. Paraffin-embedded liver sections were examined under a confocal microscope. Scale bar, 20 μm.

Finally, we determined the localization of GFAP and cit-GFAP in the livers of the hepatic fibrosis model. As shown in Fig 6A, GFAP and cit-GFAP immunoreactivity were more intensely stained in the bile duct area of hepatic fibrosis model mice than in sham-operated controls, and these results were correlated with the expression patterns of the western blot analysis. To further determine the cellular localization of cit-GFAP, we performed a double immunofluorescence analysis of the livers from sham-operated and BDL mice using cit-GFAP with total GFAP, α-SMA, CK19, SE-1 or F4/80 (Fig 6B). Interestingly, cit-GFAP was colocalized with α-SMA-positive or CK19-positive cells in the livers of BDL mice. These results indicated that cit-GFAP is localized in HSCs and cholangiocytes. However, cit-GFAP was not localized in Kupffer cells (F4/80) or hepatic sinusoidal endothelial cells (SE-1). Taken together, these findings indicated that upregulated GFAP and abnormal accumulation of cit-GFAP may play an important role in activated HSCs and portal fibroblasts of the livers of BDL mice.

## Discussion

In the present study, we first demonstrated that protein citrullination, along with upregulation and an increase in PAD, occur in a widely used BDL mouse model of hepatic fibrosis. We used a mouse model with characteristically induced bile duct injury, thus exhibiting cholangiocyte damage and increased ductular reactions.

It has been demonstrated that PAD is immunohistochemically stained only in human liver biopsy samples from chronic hepatitis and hepatic fibrosis but not from normal liver and that the degree of hepatic fibrosis and hepatic inflammation is associated with the intensity of PAD immunochemical staining [25]. Other study reported that the serum concentration of anti-modified citrullinated vimentin (anti-MCV) antibodies was significantly higher in patients with hepatic fibrosis than in patients with no hepatic fibrosis [26]. Vassiliadis E et al. demonstrated increased circulating levels of citrullinated and matrix metalloproteinases (MMP)-degraded vimentin (VICM) in a mouse model of hepatic fibrosis and in patients with early hepatic fibrosis associated with HCV and nonalcoholic fatty liver disease [27]. Additional finding demonstrates that VICM level is correlated with the level of PAD in a rat model of hepatic fibrosis in the presence of a pan-PAD inhibitor. The treatment with PAD inhibitor led to significant decrease in VCIM level in rat livers of CCl4-induced hepatic fibrosis compared to controls [28].

Although PAD and citrullinated protein might be expressed and influenced in hepatic inflammation or fibrosis, the expression and role of PAD and citrullinated protein are unclear in hepatic fibrosis. We observed that PAD2 with α-SMA or CK19 were partially merged in the mouse livers of hepatic fibrosis compared with controls (Fig 3). These findings indicate that PAD2 is predominantly found in activated HSCs and activated portal fibroblasts and that PAD influences the process of hepatic fibrosis in cholangiocytes or ductular cells as well as HSCs. In TAA-induced hepatic fibrosis, the percentage of colocalization of PAD2 with α-SMA or CK19 was relatively lower than BDL-induced hepatic fibrosis (S2 Fig). This is probably because the experimental period of the TAA-induced hepatic fibrosis model was not long enough to develop liver fibrosis compared to BDL model.

Increased citrullinated proteins and/or upregulated PADs have been reported in various human diseases, including rheumatoid arthritis [29, 30], cancer [30, 31], psoriasis [19], Alzheimer’s disease [21, 22], multiple sclerosis [32, 33]. Accumulation of citrullinated proteins is associated with disease development or progression; therefore, they can be considered useful diagnostic markers and therapeutic targets for various human diseases. Because it has been postulated that citrullination has an important effect on the structure and function of proteins, increased expression of PAD2 and citrullinated proteins in activated HSCs and portal fibroblasts could influence the process of hepatic fibrosis.

Makrygiannakis et al. [34] reported that increased citrullination was found in several inflammatory tissues, suggesting that this modification is inflammation dependent process rather than disease-related event. Citrullination may occur upon the release of pro-inflammatory stimuli and increased cell death, which induce PAD activation leading to accumulation of citrullinated proteins in inflamed tissues [30]. Recently, Samara KD et al. reported that citrullination is an important process in both idiopathic pulmonary fibrosis and the rheumatoid lung, strongly implicating neutrophils activation [35]. Therefore, PAD-mediated citrullination may influence the several pathways in hepatic inflammation and fibrosis.

GFAP is regulated by multiple posttranslational modifications [33, 36–38]. Citrullinated GFAP and vimentin have been noted in Alzheimer’s disease patient. Interestingly, GFAP was increased in activated HSCs and GFAP-expressing HSCs, and myofibroblasts accumulated in and around hepatic fibrosis [11], although vimentin was increased in hepatic fibrosis but not in activated HSCs. Among the important structural proteins in hepatic fibrosis, GFAP is highly susceptible to PAD2. Therefore, we focused on citrullination of GFAP in activated HSCs, and we first demonstrate that GFAP generally increases as liver fibrosis progresses, which also increases cit-GFAP. GFAP was previously found to be expressed in ductular reactions in the livers of mice with choline-deficient ethionine-supplemented diet-induced mouse liver injury [39], and GFAP is expressed in cholangiocytes in the livers of mice with TAA-induced hepatic fibrosis [40].

Although the significance of the increase in cit-GFAP in hepatic fibrosis remains unclear, it was reported that treatment with a PAD inhibitor decreased hepatic fibrosis in an animal experimental model [28], and therefore, the citrullination of GFAP may be associated with the progression of hepatic fibrosis. We suggest that additional studies using PAD inhibitors are needed in this regard and to identify citrullinated proteins related to hepatic fibrosis, which are not yet known. Moreover, a study of the changes and roles of PAD and citrullinated proteins in hepatic fibrosis of human samples is warranted.

In conclusion, we demonstrated for the first time that PAD2 and citrullinated proteins, especially cit-GFAP, are increased in hepatic fibrosis. Our findings implicate that the abnormal accumulation of cit-GFAP may play an important role in the progression of liver fibrosis and that cit-GFAP can be used as a biomarker and a therapeutic target for liver disease.

## Supporting information

S1 Fig Comparison of PAD2 activity and expression levels of F95, PAD2, cit-GFAP and GFAP in livers from TAA-treated mice and controls. (A) Densitometric analysis of PAD2 activity demonstrated that the liver tissue of TAA-treated mice (n = 9) showed increased PAD2 activity compared with controls (n = 9). *P <0.05. (B) The expression level of F95, PAD2, cit-GFAP and GFAP in the liver tissues of control and TAA-treated mice was analyzed by western blot. Densitometric analysis of PAD2 and F95 demonstrated that the liver tissue of TAA-treated mice (n = 9) showed increased protein expression compared with controls (n = 9). **P <0.01.
(TIF)

S2 Fig Double immunofluorescence of PAD2 and α-SMA or CK19 in livers from TAA-treated mice. Cryosections were examined under a confocal microscope. PAD2 immunoreactivity was increased in the livers of TAA-treated mice. Alpha-SMA immunoreactivity was also increased in the livers of TAA-treated mice. PAD2 was partially colocalized with α-SMA-positive cells in and around the bile duct area. And CK19 immunoreactivity was increased in the livers of TAA-treated mice. PAD2 was also colocalized with CK19-positive cells in and around the bile duct area.
(TIF)

S3 Fig The original uncropped and unadjusted blots in the Figs 2, 4, 5 and S1 Fig. (PDF)

## Abbreviations

HSC: hepatic stellate cells
SMA: smooth muscle actin
GFAP: glial fibrillary acidic protein
IF: intermediate filament
PAD: peptidylarginine deiminase
BDL: bile duct ligation
RT: room temperature
GAPDH: glyceraldehyde-3-phosphate dehydrogenase
Col1a1: collagen type I
CK: cytokeratin

## References

[1] Bataller R, Brenner DA. Liver fibrosis. *J Clin Invest* 2005;115(2):209–18. 10.1172/JCI24282 15690074 PMC546435

[2] Iredale JP. Models of liver fibrosis: exploring the dynamic nature of inflammation and repair in a solid organ. *J Clin Invest* 2007;117(3):539–548. 10.1172/JCI30542 17332881 PMC1804370

[3] Suk KT, Baik SK, Yoon JH, Cheong JY, Paik YH, Lee CH, et al Revision and update on clinical practice guideline for liver cirrhosis. Korean J Hepatol 2012;18(1):1–21. 10.3350/kjhep.2012.18.1.1 22511898 PMC3326994

[4] Bonis PA, Friedman SL, Kaplan MM. Is liver fibrosis reversible? *N Eng J Med* 2001;344(6):452–4. 10.1056/NEJM200102083440610 11172184

[5] Higashi T, Friedman SL, Hoshida Y. Hepatic stellate cells as key target in liver fibrosis. Adv Drug Deliv Rev 2017;121:27–42. 10.1016/j.addr.2017.05.007 28506744 PMC5682243

[6] Knittel T, Kobold D, Iscaglia F, Saile B, Neubauer K, Mehde M, et al Localization of liver myofibroblasts and hepatic stellate cells in normal and diseased rat livers: distinct roles of (myo-)fibroblast subpopulations in hepatic tissue repair. Histochem Cell Bio 1999;112(5):387–401. 10603079 10.1007/s004180050421

[7] Desmouliere A, Darby IA, Gabbiani G. Normal and pathologic soft tissue remodeling: role of the liver myofibroblast with special emphasis on liver and kidney fibrosis. Lab Invest 2003;83(12):1689–707. 14691287 10.1097/01.lab.0000101911.53973.90

[8] Guyot C, Lepreux S, Combe C, Doudnikoff E, Biolac-Sage P, Balabaud C, et al Hepatic fibrosis and cirrhosis:the (myo)fibroblastic cell subpopulations involved. *Int J Biochem Cell Biol* 2006;38(2): 135–151. 10.1016/j.biocel.2005.08.021 16257564

[9] Yang Z, Wang KK. Glial fibrillary acidic protein: from intermediate filament assembly and gliosis to neurobiomarker. Trends Neurosci 2015;38(6):364–74. 10.1016/j.tins.2015.04.003 25975510 PMC4559283

[10] Cassiman D, Libbrecht L, Desmet V, Denef C, Roskams T. Hepatic stellate cell/myofibroblast subpopulations in fibrotic human and rat livers. J Hepatol 2002;36(2):200–9. 11830331 10.1016/s0168-8278(01)00260-4

[11] Niki T, De Bleser PJ, Xu G, Van Den Berg K, Wisse E, Geerts A. Comparison of glial fibrillary acidic protein and desmin staining in normal and CCl4-induced fibrotic rat livers. Hepatology 1996;23(6):1538–1545. 10.1002/hep.510230634 8675175

[12] Tennakoon AH, Izawa T, Wijesundera KK, Golbar HM, Tanaka M, Ichikawa C, et al Characterization of glial fibrillary acidic protein-expressing hepatic stellate cells and myofibroblasts in thioacetamide-induced rat liver injury. ExpToxicolPathol 2013;65(7-):1159–71. 10.1016/j.etp.2013.05.008 23806769

[13] Tennakoon AH, Izawa T, Wijesundera KK, Murakami H, Katou-Ichikawa C, Tanaka M, et al Immunochemical characterization of glial fibrillary acidic protein-expressing cells in a rat liver cirrhosis model induce by repeated injections of thioacetamide. ExpToxicol Pathol 2014;67(1):53–63. 10.1016/j.etp.2014.09.008 25446803

[14] Ishigami A, Maruyama N. Importance of research on peptidylarginine deiminase and citrullinated proteins in age-related disease. GeriatriGerontolInt 2010;10(Suppl 1):S53–58. 10.1111/j.1447-0594.2010.00593.x 20590842

[15] Jang B, Ishigami A, Maruyama N, Carp RI, Kim YS, Choi EK. Peptidylarginine deiminase and protein citrullination in prion disease: strong evidence of neurodegeneration. Prion 2013;7(1):42–46. 10.4161/pri.22380 23022892 PMC3609049

[16] Lamensa JW, Moscarello MA. Deimination of human myelin basic protein by a peptidylarginine deiminase from bovine brain. J Neurochem 1993;61(3):987–996. 7689646 10.1111/j.1471-4159.1993.tb03612.x

[17] Tarcsa E, Marekov LN, Mei G, Melino G, Lee S-C, Steinert PM. Protein unfolding by peptidylarginine deiminase. Substrate specificity and structural relationships of the natural substrates trichohyalin and filaggrin. J Biol Chem 1996;71(48):30709–30716. 10.1074/jbc.271.48.30709 8940048

[18] Vossennaar ER, Zendman AJ, van Venrooij WJ, Pruijn GJ. PAD, a growing family of citrullinating enzymes: genes, features and involvement in disease. Bioessays 2003;25(11):1106–1118. 10.1002/bies.10357 14579251

[19] Méchin MC, Sebbag M, Arnaud J, Nachat R, Foulquier C, Adoue V, et al Update on peptidylarginine deiminases and deimination in skin physiology and severe human disease. Int J Cosmet Sci 2007;29(3):147–168. 10.1111/j.1467-2494.2007.00377.x 18489346

[20] Asaga H, Senshu T. Combined biochemical and immunocytochemical analyses of postmortem protein deamination in the rat spinal cord. Cell BiolInt 1993;17(5):525–532. 10.1006/cbir.1993.1094 8339070

[21] Ishigami A, Ohsawa T, Hiratsuka M, Taquchi H, Kobayashi S, Saito Y, et al Abnormal accumulation of citrullinated proteins catalyzed by peptidylarginine deiminase in hippocampal extracts from patients with Alzheimer’s disease. J Neurosci Res 2005;80(1):120–128. 10.1002/jnr.20431 15704193

[22] Ishigami A, Masutomi H, Handa S, Nakamura M, Nakya S, Uchida Y, et al Mass spectrometric identification of citrullinated sites and immunohistochemical detection of citrullinated glial fibrillary acidic protein in alzheimenr’s disease brains. J Neurosci Res 2015;93(11):1664–1674. 10.1002/jnr.23620 26190193

[23] Shimada N, Handa S, Uchida Y, Fukuda M, Maruyama N, Asaga H, et al Developmental and age-related changes of peptidylarginine deiminase 2 in the mouse brain. J Neurosci Res 2010;88(4):798–806. 10.1002/jnr.22255 19830834

[24] Watanabe K, Akiyama K, Hikichi K, Ohtsuka R, Okuyama A, Senshu T. Combined biochemical and immunochemical comparison of peptidylarginine deiminases present in various tissues. BiochimBiophysActa 1988;966(3):375–383. 10.1016/0304-4165(88)90088-8 3416014

[25] Abdeen SM, Olusi SO. Peptidyl arginine deiminase: A novel immunohistochemical marker for liver fibrosis in patients with chronic hepatitis. Acta Histochem 2010;112(6):592–603. 10.1016/j.acthis.2009.06.007 19836826

[26] Abdeen S, Olusi SO, George S. Serum anti-modified citrullinated vimentin antibody concentration is associated with liver fibrosis in patients with chronic hepatitis. Hepat Med 2011;3:13–8. 10.2147/HMER.S17039 24367216 PMC3846543

[27] Vassiliadis e Oliveira CP, Alvares-da-Silva MR, Zhang C, Carrilho FJ, Stefano JT, et al Circulating levels of citrullinated and MMP-degraded vimentin (VCIM) in liver fibrosis related pathology. Am J Transl Res 2012;4(4):403–414. 23145208 PMC3493028

[28] Vassiliadis E, Veidal SS, Kristiansen MN, Hansen C, Jorgensen M, Leeming DJ, et al Peptidyl arginine deiminase inhibitor effect on hepatic fibrogenesis in a CCl4 pre-clinical model of liver fibrosis. Am J Transl Res 2013;5(4):465–469. 23724169 PMC3665919

[29] Luban S, Li ZG. Citrullinated peptide and its relevance to rheumatoid arthritis: update. Int J Rheum Dis 2010;13(4):284–287. 10.1111/j.1756-185X.2010.01553.x 21199462

[30] Gudamnn NS, Hansen NU, Jensen AC, Karsdal MA, Siebuhr AS. Biological relevance of citrullination: diagnostic, prognostic and therapeutic options. Autoimmunity 2015;48(3):73–79. 25520183 10.3109/08916934.2014.962024

[31] Guo W, Zheng Y, Xu B, Ma F, Li C, Zhang X, et al Investigating the expression, effect and tumorigenic pathway of PADI2 in tumors. Onco Targets Ther 2017;10:1475–1485. 10.2147/OTT.S92389 28331341 PMC5352236

[32] Moscarello MA, Mastronardi FG, Wood DD. The role of citrullinated proteins suggests a novel mechanism in the pathogenesis of multiple sclerosis. Neurochem Res 2007;32(2):251–256. 10.1007/s11064-006-9144-5 17031564 PMC1794624

[33] Nicholas AP, Sambandam T, Echols JD, Tourtellotte WW. Increased citrullinated glial fibrillary acidic protein in secondary progressive multiple sclerosis. J Comp Neruol 2004;473(1):128–136. 10.1002/cne.20102 15067723

[34] Makrygiannakis D, at Klint E, Lundberg IE, Lofberg R, Ulfgren AK Klareskog L, et al Citrullination is an inflammation-depedent process. Ann Rhuem Dis 2006;65(9):1219–1222. 10.1136/ard.2005.049403 PMC1798285 16540548

[35] Samara KD, Trachalaki A, Tsitoura E, Koutsopoulos AV, Lagoudaki ED, Lasithiotaki I, et al Upregulation of citrullination pathway: from autoimmune to idiopathic lung fibrosis. Respir Res 2017;18(1):218 10.1186/s12931-017-0692-9 29287593 PMC5747943

[36] Ralton JE, Lu X, Hutcheson AM, Quinlan RA. Identification of two N-terminal non-alpha-helical domain motifs important in the assembly of glial fibrillary acidic protein. J Cell Sci 1994;107(7):1935–1948. 7983160 10.1242/jcs.107.7.1935

[37] Inagaki M, Nishi Y, Nishizawa K, Matsuyama M, Sato C. Site-specific phosphorylation induces disassembly of vimentin filaments in vitro. Nature 1987;328(6131):649–652. 10.1038/328649a0 3039376

[38] Nicholas AP, King JL, Sambandam T, Echols JD, Gupta KB, Mclnnis C, et al Immunohistochemical localization of citrullinated proteins in adult rat brain. J Comp Neurol 2003;459(3):251–266. 10.1002/cne.10607 12655508

[39] Ueberham E, Böttger J, Ueberham U, Grosche J, Gebhardt R. Response of sinusoidal mouse liver cells to choline-deficient ethionine-supplemented diet. Comp Hepatol 2010;9:8 10.1186/1476-5926-9-8 20942944 PMC2964607

[40] Tennakoon A, Izawa T, Wijesundera KK, Murakami H, Katou-Ichikawa C, Tanaka M, et al Analysis of glial fibrillary acidic protein-expressing ductular cells in a rat liver cirrhosis model induced by repeated injections of thioacetamide. Exp Mol Pathol 2015;98(3):476–485. 10.1016/j.yexmp.2015.03.010 25758201

