# Supplementary figures and images for: Accumulation of citrullinated glial fibrillary acidic protein in a mouse model of bile duct ligation-induced hepatic fibrosis
Source: PLoS One. 2018 Aug 2;13(8):e0201744. doi: 10.1371/journal.pone.0201744 (PMC6072123; doi:10.1371/journal.pone.0201744)

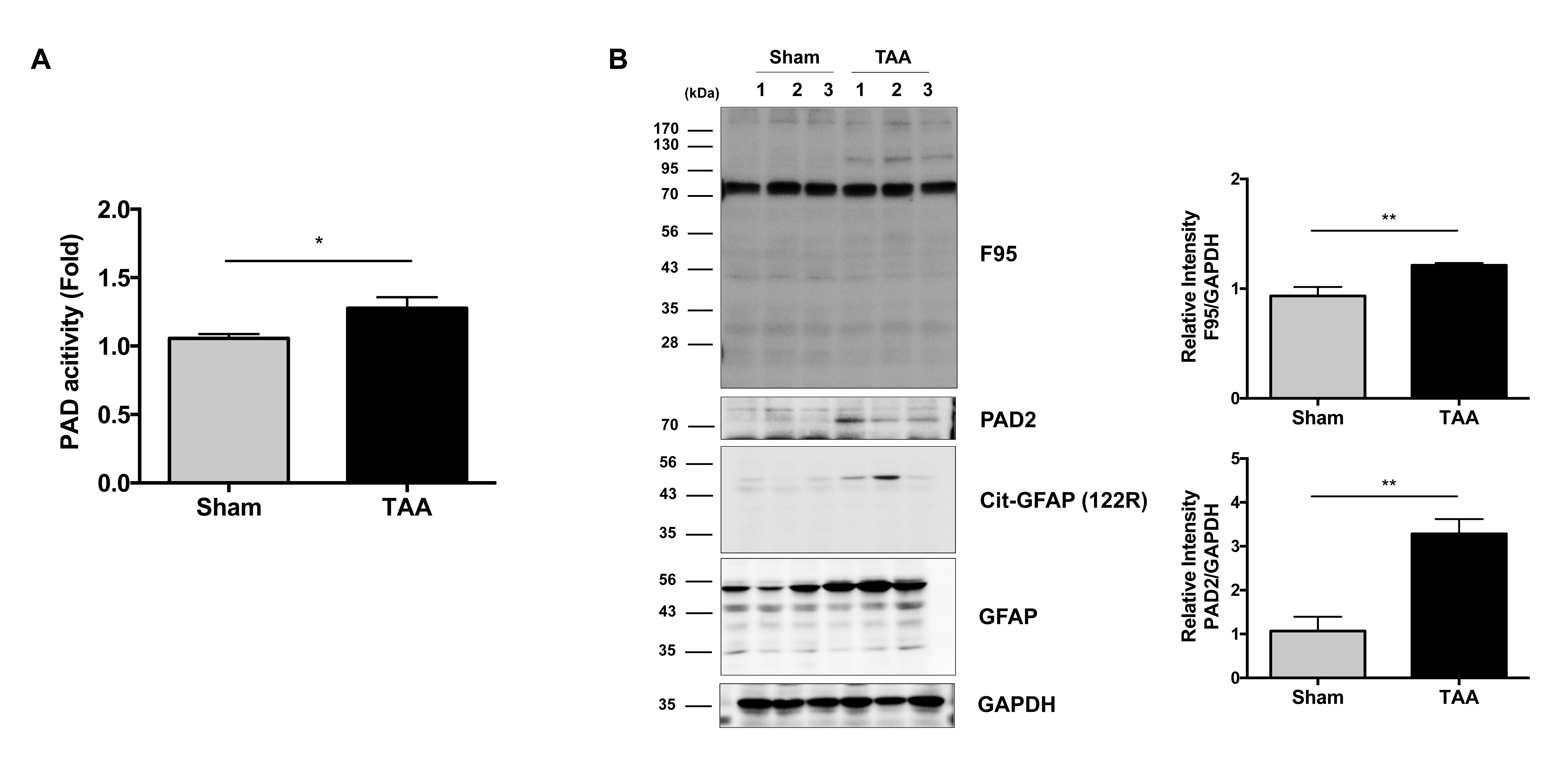

Supplement: S1 Fig — (A) Densitometric analysis of PAD2 activity demonstrated that the liver tissue of TAA-treated mice (n = 9) showed increased PAD2 activity compared with controls (n = 9). *P <0.05. (B) The expression level of F95, PAD2, cit-GFAP and GFAP in the liver tissues of control and TAA-treated mice was analyzed by western blot. Densitometric analysis of PAD2 and F95 demonstrated that the liver tissue of TAA-treated mice (n = 9) showed increased protein expression compared with controls (n = 9). **P <0.01. (TIF) [file pone.0201744.s001.tif]

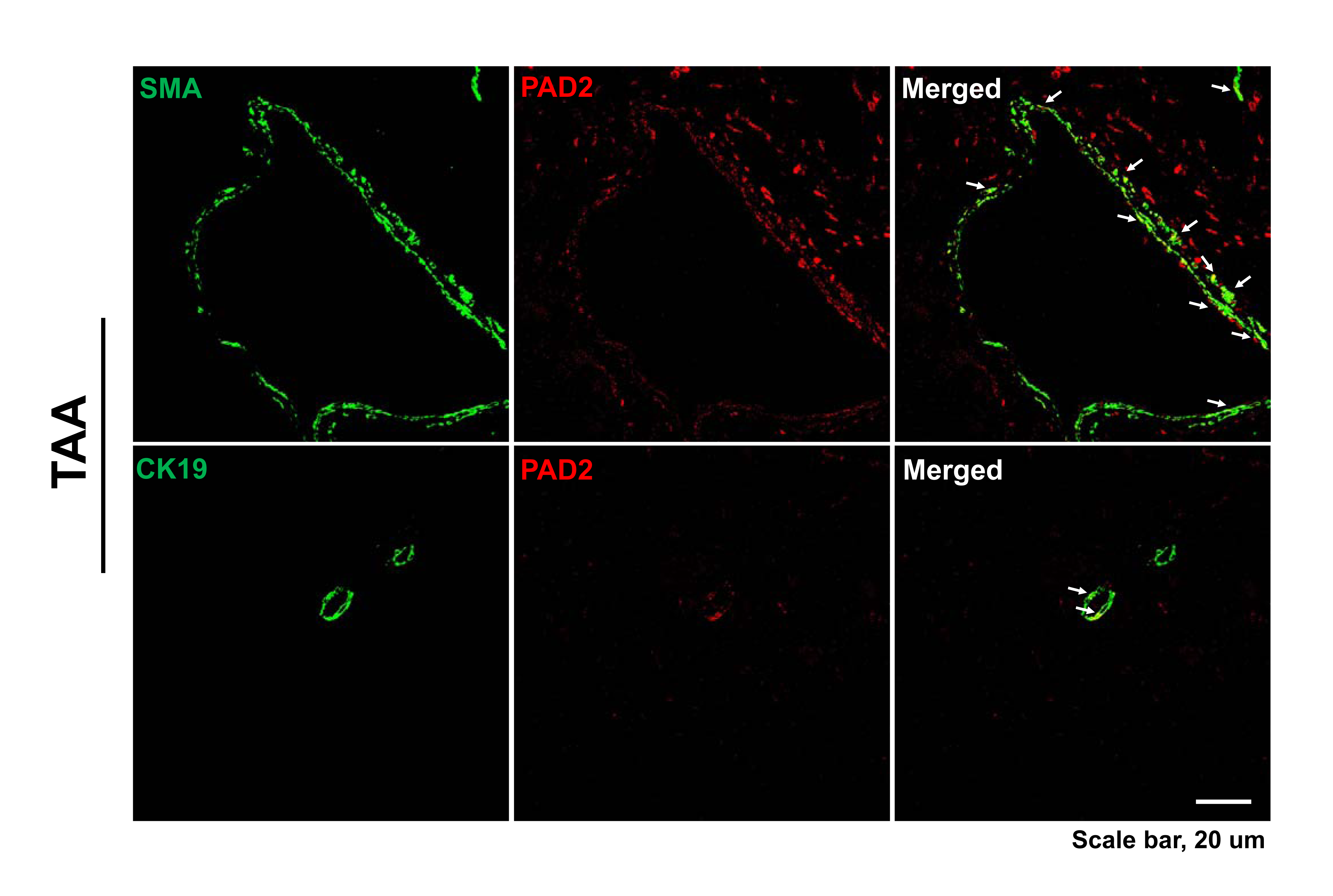

Supplement: S2 Fig — Cryosections were examined under a confocal microscope. PAD2 immunoreactivity was increased in the livers of TAA-treated mice. Alpha-SMA immunoreactivity was also increased in the livers of TAA-treated mice. PAD2 was partially colocalized with α-SMA-positive cells in and around the bile duct area. And CK19 immunoreactivity was increased in the livers of TAA-treated mice. PAD2 was also colocalized with CK19-positive cells in and around the bile duct area. (TIF) [file pone.0201744.s002.tif]

Figure 2

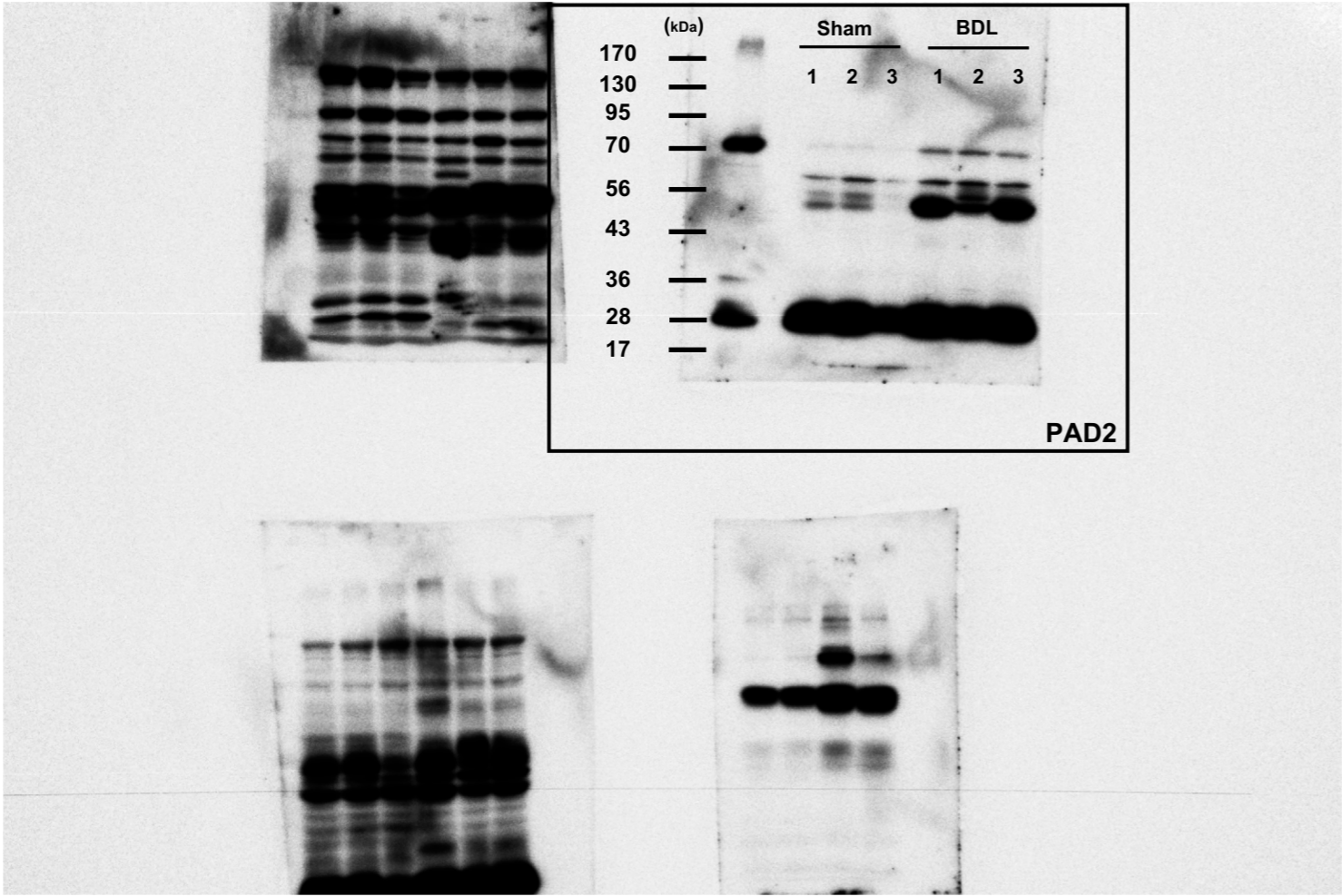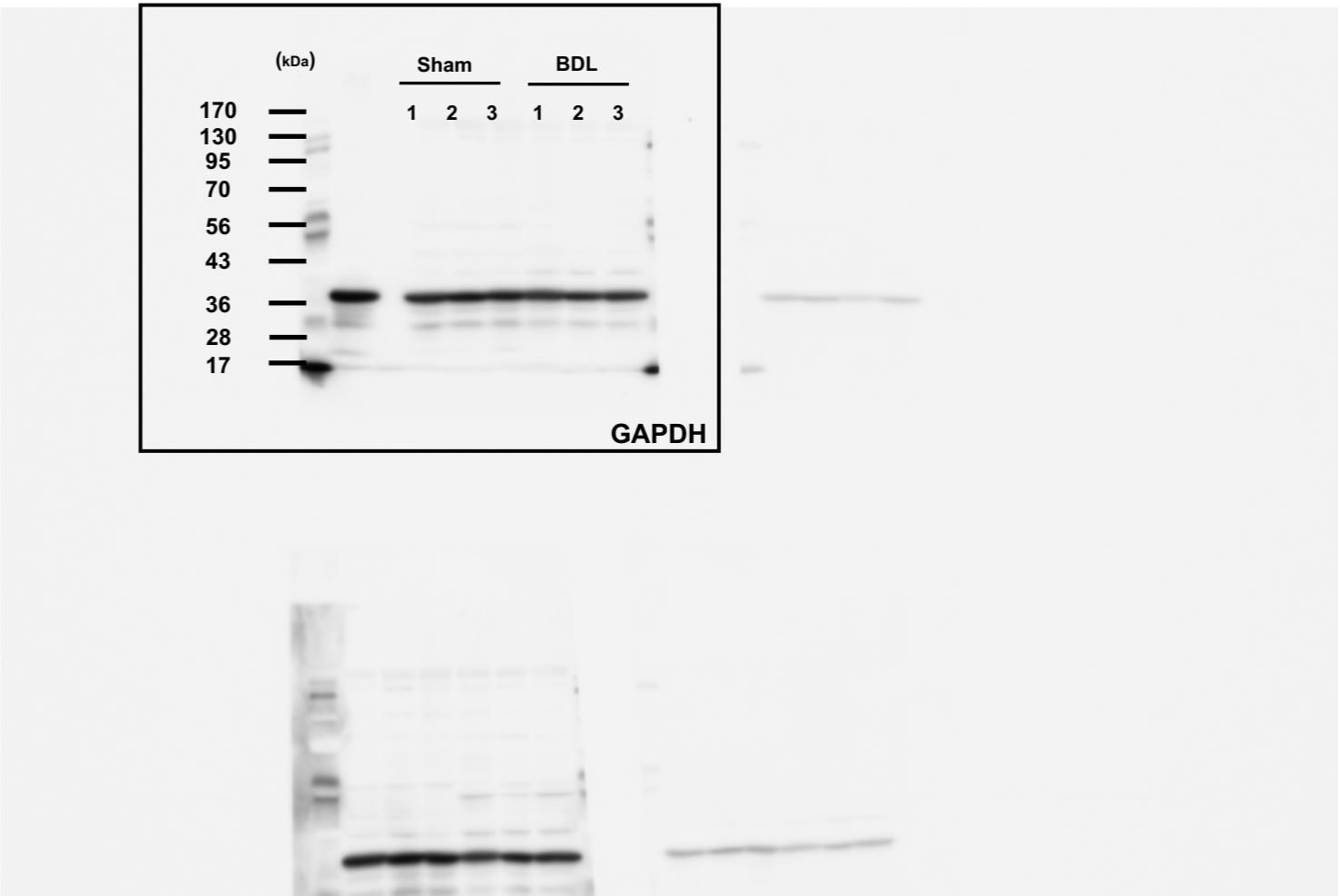

Figure 4

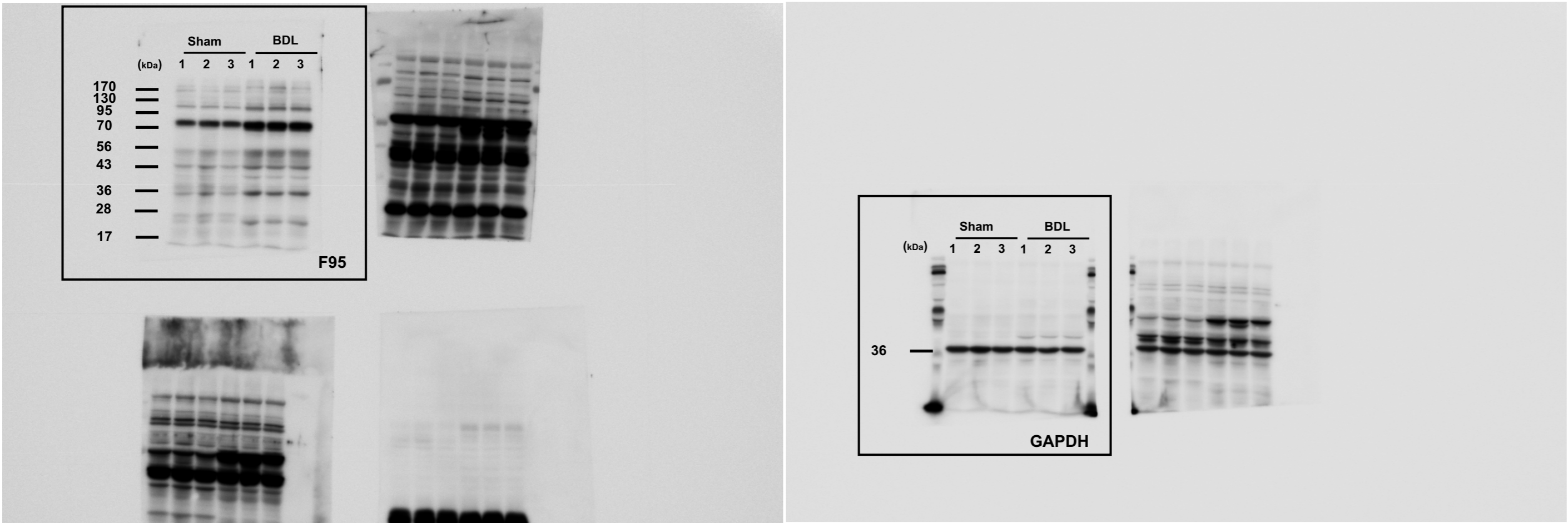

Figure 5

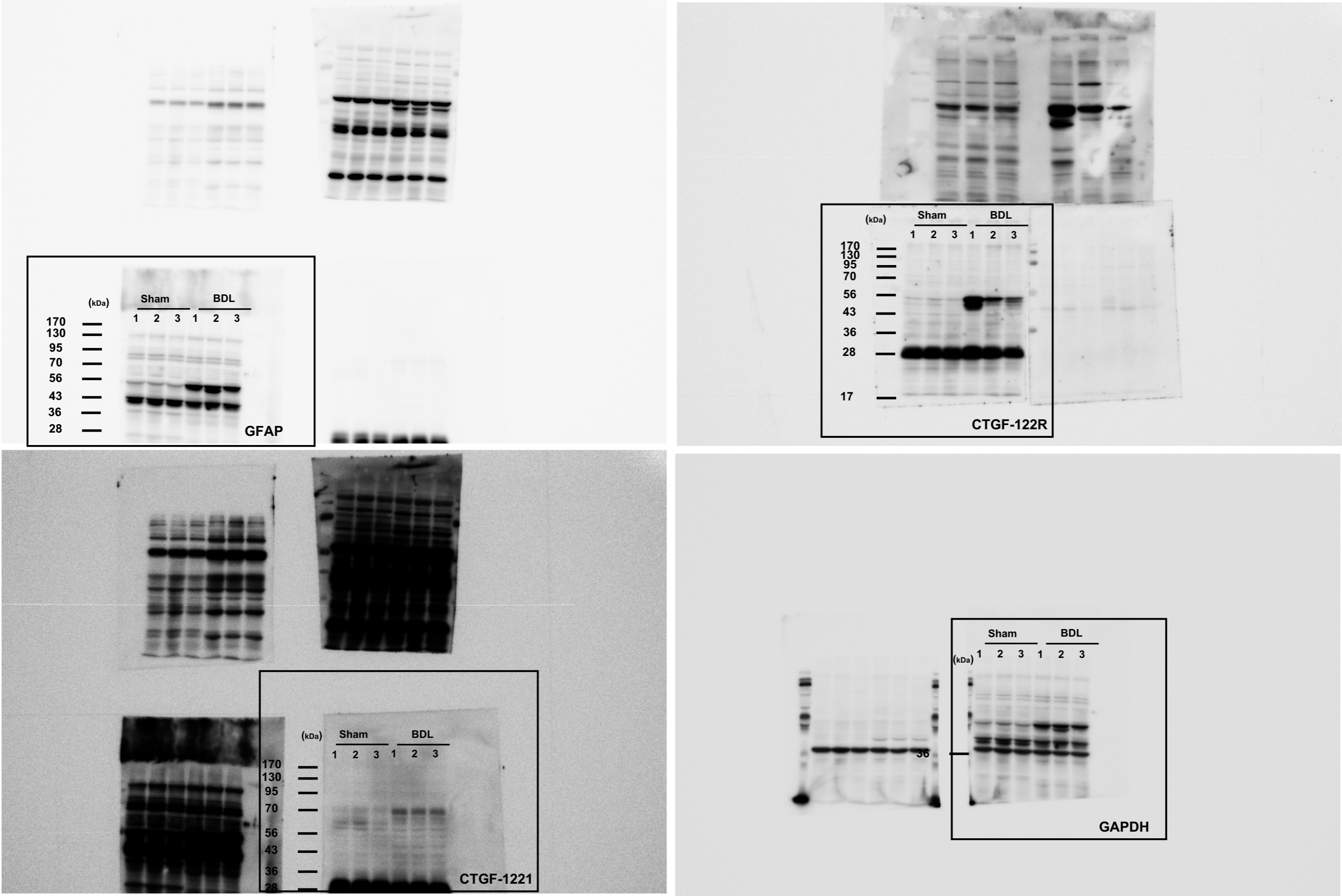

S1 Figure

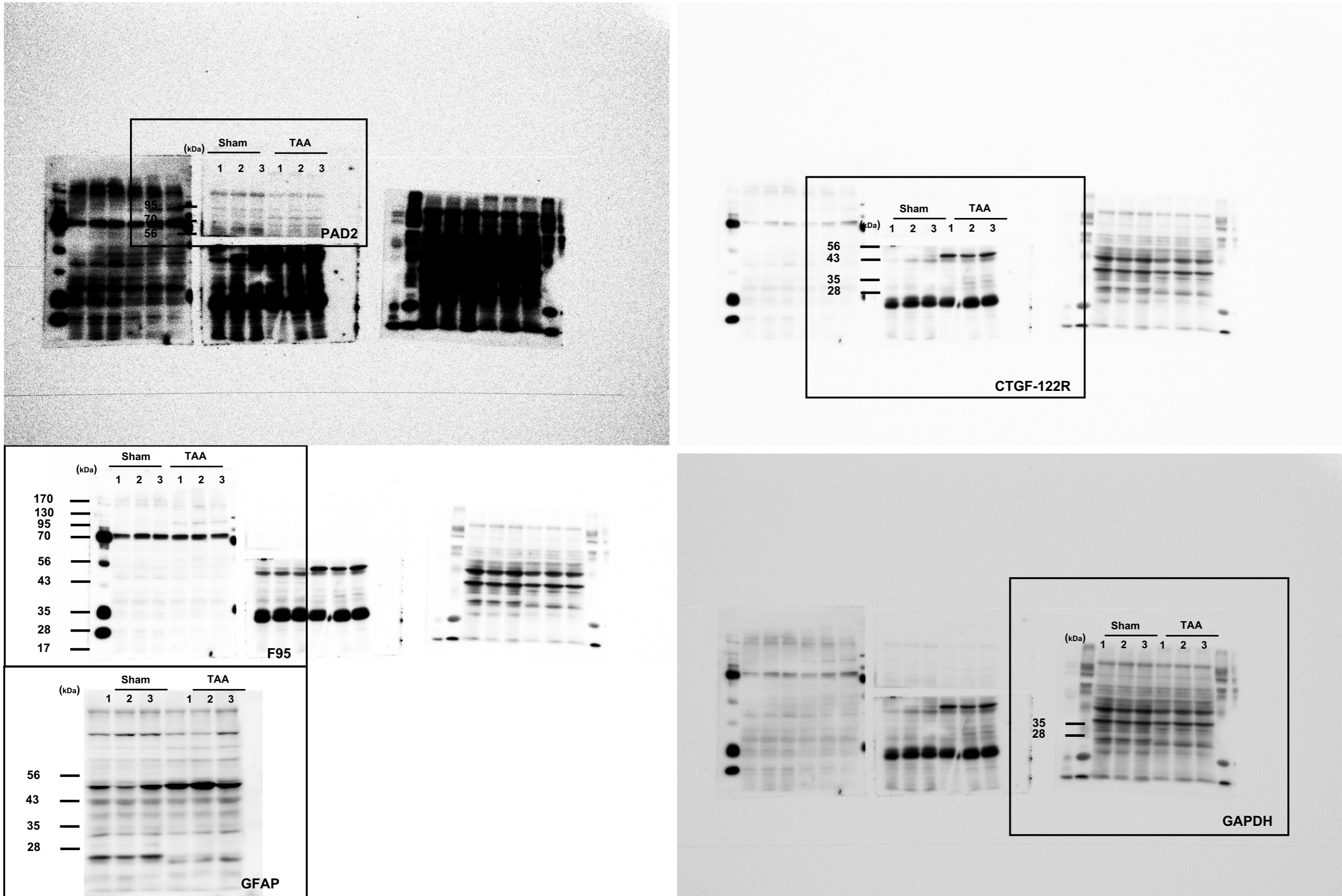

Supplement: S3 Fig — (PDF) [file pone.0201744.s003.pdf]
